# Greco-Roman mineral (litho)therapeutics and their relationship to their microbiome: The case of the red pigment *miltos*

**DOI:** 10.1016/j.jasrep.2018.07.017

**Published:** 2018-12

**Authors:** E. Photos-Jones, C.W. Knapp, D. Venieri, G.E. Christidis, C. Elgy, E. Valsami-Jones, I. Gounaki, N.C. Andriopoulou

**Affiliations:** a Analytical Services for Art and Archaeology (Ltd), Glasgow G12 8JD, UK; b Archaeology, School of Humanities, University of Glasgow, Glasgow G12 8QQ, UK; c Civil and Environmental Engineering, University of Strathclyde, Glasgow G1 1XQ, UK; d School of Environmental Engineering, Technical University of Crete, 73100 Chania, Greece; e School of Geography, Earth and Environmental Sciences, University of Birmingham, Edgbaston, Birmingham B15 2TT, UK; f School of Mineral Resources Engineering, Technical University of Crete, 73100 Chania, Greece

**Keywords:** Greco-Roman mineral medicinals, Lithotherapeutics, *Miltos*, Minerals, Microbiota, Nanoparticles, Bioactivity

## Abstract

This paper introduces a holistic approach to the study of Greco-Roman (G-R) lithotherapeutics. These are the minerals or mineral combinations that appear in the medical and scientific literature of the G-R world. It argues that they can best be described not simply in terms of their bulk chemistry/mineralogy but also their ecological microbiology and nanofraction component. It suggests that each individual attribute may have underpinned the bioactivity of the lithotherapeutic as an antibacterial, antifungal or other. We focus on *miltos*, the highly prized, naturally fine, red iron oxide-based mineral used as a pigment, in boat maintenance, agriculture and medicine. Five samples (four geological (from Kea, N. Cyclades) and one archaeological (from Lemnos, NE Aegean)) of *miltos* were analyzed with physical and biological science techniques. We show that: a. Kean *miltos* and Lemnian earth/*miltos* must have been chemically and mineralogically different; b. Lemnian *miltos* must have been more effective as an antibacterial against specific pathogens (Gram + and Gram − bacteria) than its Kean counterpart; c. two samples of Kean *miltos*, although similar, chemically, mineralogically and eco-microbiologically (phylum/class level), nevertheless, displayed different antibacterial action. We suggest that this may constitute proof of microbial ecology playing an important role in effecting bioactivity and, interestingly, at the more specific genus/species level. From the perspective of the historian of G-R science, we suggest that it may have been on account of its bioactivity, rather than simply its 'red-staining' effect, that *miltos* gained prominent entry into the scientific and medical literature of the G-R world.

## Highlights

• Greco-Roman mineral medicinals have a long, successful track record of being therapeutic and having diverse applications.
• *Miltos* was a prized red (iron oxide) pigment but its applications were also in medicine, agriculture and ship maintenance.
• *Miltos* is studied holistically: its mineralogy nano-fraction component, ecological microbiology and antibacterial activity.
• Even minor modifications in *miltos*’ mineral+microorganism signature can alter its bioactivity and commercial applications.
• We suggest that in the G-R medical/scientific literature the bioactivity of *miltos* was more important than its red colour.

## 1 Introduction

Greco-Roman (G-R) medical and other texts (for example, Theophrastus *On Stones*; Dioscorides *De Materia Medica* Book V; Pliny *Natural History* Book 35) list not only plants but also minerals and animal products as therapeutic agents, albeit the last two in fewer numbers. These therapeutic materials derived from specific localities and had many and diverse applications in fields not obviously related to medicine, such as pigments, washing powders, textile treatment, or agriculture. Curiously, the basis on which minerals built up their multiple market applications has largely gone unquestioned by modern scholarship; perhaps it was deemed idiosyncratic of the G-R world. Nevertheless, questions remain: was the *same* mineral/mineral combination extracted from each locality and used in each market application and how was ‘same’ to be assessed?

The study of the minerals industry of the G-R world is still in its infancy. This is primarily on account of the inherent difficulty in attributing what are largely descriptive terms to inorganic substances with precise chemical compositions and crystal structure. G-R minerals nomenclature incorporates both the natural (the raw material) and the processed (the marketable product) and does not always separate between the two. In other words, the G-R texts present the reader with the mineral's commercial or group name, meaning that many different mineral varieties were traded and exchanged under the same name. In an attempt to navigate around this network of complex information, scholars have traditionally assumed a best-fit approach. For example, *lithargyros* has been translated as ‘litharge’, meaning the mineral PbO. That is despite Dioscorides' (De Materia Medica V.87) and Pliny's (Natural History 33.35) efforts to make it clear to their readers that *lithargyros* existed in three varieties, acknowledging, implicitly, both different method of manufacture as well as a different chemical product.

We therefore argue that in order to understand the nature of the G-R minerals and the properties underpinning their diverse applications, they should be studied ‘at source’ and based on geological samples. This does not mean that we are confident that ancient miners worked the exact same mineral as the ones we have sampled. Rather, when ancient miner and today's geoarchaeologist confront the same type of ‘deposit’, in the same locality, there is sufficient confidence that ancient and modern observations will coincide, albeit expressed differently. Having established the need to examine minerals at source, it follows that there is a need to put in place a methodology for their characterisation. In this paper we argue that, apart from their mineralogy and geochemistry, it is vital to study their microbial ecology as well because of the link between microorganisms and their environment, as discussed below.

**Fig. 1:**
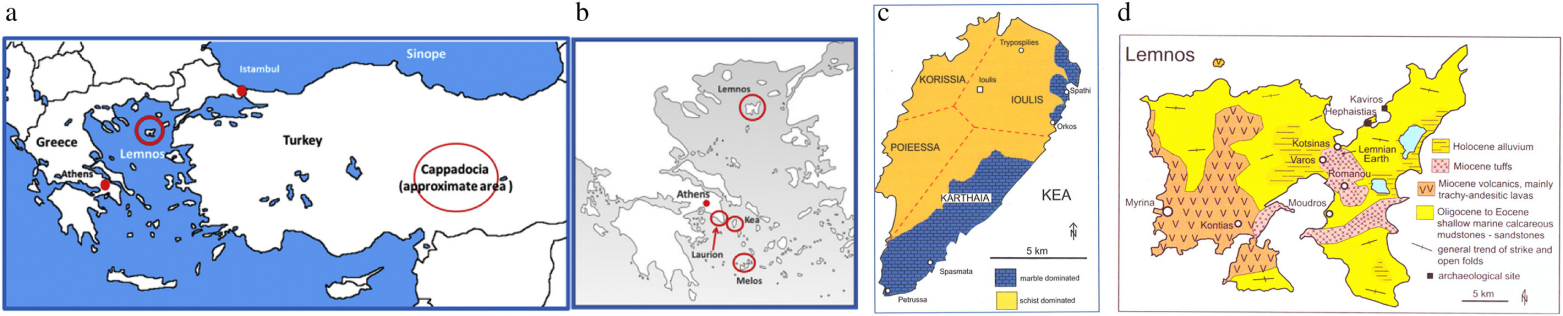
a. Map of Greece and Turkey with localities mentioned in the text. b Map of Attica and the Aegean with localities mentioned in the text. c Map of Kea with approximate outline of the realm of each of the four city states in the 4th century BC and localities of extraction of *miltos* (after Photos-Jones and Hall, 2011, Fig. 45). d. map of Lemnos showing locality of Kotsinas, place of extraction of Lemnian Earth (after Photos-Jones and Hall, 2011, Fig. 31). Petroussa is the area between localities 4 and 5 on this map.).

We focus here on a single G-R mineral, *miltos* (*ruddle*, *L. rubrica*), the naturally occurring, highly prized, fine-particled red iron oxide ‘pigment’ of antiquity (Cherry et al., 1991; Photos-Jones et al., 1997; Lytle, 2013). *Miltos* first appears in the literature in the 4th century BC (Caley and Richards, 1956 translation of Theophrastus *On Stones*, 52–54) but it actually features in early writing (Linear B script) on Mycenaean clay tablets dated to the late 2nd millennium BC (Blakolmer, 2004). *Miltos*, unlike ‘common’ ochres (Theophrastus *On Stones*, 51) or ‘*haematitis lithos*’ (hematite) (Dioscorides *De Materia Medica* V.114), had very specific places of origin, including the Cycladic island of Kea, the island of Lemnos in the NE Aegean and Sinope/Cappadocia in Turkey (Fig. 1a and b). The Sinopic *miltos* was actually produced in Cappadocia but shipped from Sinope (Theophrastus, *On Stones*, 52). *Miltos* was primarily characterised by its colour, red, which had considerable staining power (Αristophanes, *Ecclesiazousae*, 387). It had diverse applications, as a pigment, as a cosmetic,^1^ in ship maintenance,^2^ agriculture^3^ and medicine.^4^

According to Theophrastus (*On Stones*, 52), there were three *varieties* of *miltos*, differentiated on the basis of colour hue (deep red (ἐρυθρά σφόδρα), medium (μέση) and pale (ἒκλευκος), and that the medium one was used intact, i.e. with no mixing. Artificial *miltos* could be produced from heating yellow ochre in pots, but it was deemed ‘inferior’. On Kea, there is evidence for *miltos* extraction dated to the 5th–4th century BC, and in association with iron mines in the north, east and south of the island (Fig. 1c). *Miltos* could still be removed until recently, in near-powder form, from gallery walls in the same localities; many of these galleries are presently inaccessible. During our field walking survey around the island in 1996, we sampled extensively and reported that *miltos* consisted of goethite and to a lesser extent hematite and calcite (Photos-Jones et al., 1997, Table 2). We did not report on its geochemistry but noted that it must have been the natural fineness of its particle size that made it stand out, as examined by SEM-EDAX (Photos-Jones et al., 1997, Fig. 3).

Twenty years on, we revisit some of these samples with emphasis on their geochemistry and ecological microbiology. In any habitat, microorganisms and their environment are intrinsically connected. Ecological microorganisms coexist and interact with the mineral as part of a biogeochemical cycle (Haferburg and Kothe, 2008). This interaction is a two-way process since a community is sustained by its environmental conditions (e.g. temperature, pH, redox state and nutrients), and these conditions could in turn be changed by the action of its microbiome (e.g. the microorganims' morphology, storage of waste and secreted compounds). We highlight the observation that many G-R minerals and Kean or Lemnian *miltos*, in particular, would have carried their microbiota ‘from the mine to the market’ as long as no oxidative or high temperature processes intervened. Such processes would have resulted in the elimination of the organic constituent of *miltos*, as in the case of Theophrastus' reference that yellow ochre was heated, as mentioned above. The product, the synthetic red ‘*miltos*’ he deemed inferior to the natural one. And, although he does not specify what particular aspect he regards as inferior, we suggest that the high temperature firing would have certainly removed the organic component and thus would have removed its contribution to *miltos*' bioactivity.

The presence and contribution of microorganisms to the healing attributes of therapeutic minerals is well recognised today, particularly in relation to pelloids (therapeutic muds) (Gomes and Silva, 2007). However, given the complexity of these systems and for health reasons, pelloids are routinely screened for pathogens, thus restricting a fuller understanding of the mineral-microbiota interface. We suggest that appreciation of the role of this interface is critical in the study of any therapeutic mineral used in antiquity or today.

We therefore propose that the minerals of the G-R texts should not only be described in terms of their mineralogical classification, as oxides, silicates, sulphates or other, but also in terms of their microbiome which is defined as the totality of microorganisms (and their biomolecules (secondary metabolites)) associated with the minerals at source. A G-R mineral would become a therapeutic mineral (or a *lithotherapeutic*) only when incorporated within a recipe. For example, Scribonius Largus, a 1st c AD Roman doctor, uses (Sinopic) *miltos* (*ribrica Sinopidis*) in his *Compositiones Medicamentorum*, chapter 42; it is intended for ear and nostril ailments in association with other mineral substances (for example, alum and ‘burnt’ copper) and dissolved in vinegar. In another recipe, *Compositiones Medicamentorum*, chapter 170 he prescribes Lemnian rubric (*miltos*) instead again in association with other minerals (I Jocks, pers. comm; Jocks, 2013). For two other recipes (*Comp.Med.* 228 and 230), he prescribes *miltos* but specifies no particular place of origin.

**Fig. 2:**
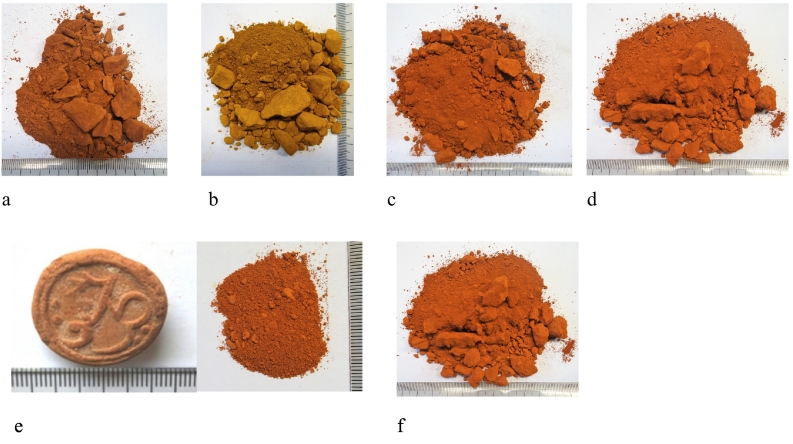
a. 730.2, Petroussa, S. Kea. b. 730.3, yellow ochre, N. Gallery, Orkos, E. Kea. c. 730.4, Trypospilies, N. Kea. d. 730.5, N. Gallery, Orkos, E. Kea. e. (left) 700.17 Red Lemnian Earth from the collection of the Pharmacy Museum of the University of Basel (after Photos-Jones et al., 2017); (right), the same sample, crushed, in preparation for analysis. f. 700.19 red clay sample from the ‘*aima tou Hephaistou*’ locality, Kotsinas, NE Lemnos. The similarity in ‘red’ across the samples is striking, although there do exist variations like in 730.2, which is a deeper shade of red than the rest. All samples have a considerable staining effect.

Five samples were selected for full investigation: four (three red and one yellow) geological samples from Kea, and one archaeological sample of red Lemnian Earth in the collection of the Pharmacy Museum of the University of Basel, Switerland dating to the Ottoman period (16th–17th centuries, dated on the basis of the ‘stamped’ Ottoman script) (Fig. 2a–e). The extraction of Lemnian Earth from the locality of Kotsinas village, NE Lemnos (Fig. 1d) is well documented from the Roman period onwards and well into the early 20th century (Jones and Hall, 2011). We have included one sample of red clay from Kotsinas for reference (Fig. 2f). The toponym of this locality of red clay is today referred to as ‘*to aima tou Hephaistou*’ (the blood of Hephaistos), since its red coloration stands out so visibly from the surrounding landscape. We cannot be confident that *red* Lemnian Earth and Lemnian *miltos* were one and the same substance (Theophrastus 52–3; Galen, XII. 169–70). For the purposes of this discussion and because we have no evidence to the contrary, we assume that they were, and so use Lemnian *miltos* and Earth interchangeably. We sampled the yellow ‘*miltos*’ for the express purpose of investigating to what extent it was different (composition, particle size etc.) to the red *miltos*. Both samples (Fig. 2b and d, respectively) come from the same mine, Orkos, E Kea. Yellow *miltos* could have been collected by Kean miners (referred to as *miltorychoi* (μιλτωρύχοι, Pollux. *Onomasticon* 7.100) for the express purpose of heating to make synthetic *miltos*, although this, of course, remains a supposition.

As mentioned earlier, in sampling and analysing geoarchaeological materials one can never be certain that the sample investigated is identical (chemically, mineralogically and microbiologically) to the minerals reported in the ancient texts. Nevertheless, our work on different G-R minerals has focused on the development of a methodology for ‘translating’ observations and processes described in the ancient texts into modern terminology by combining the documentary and archaeological record and from science-based evidence (Photos-Jones and Hall, 2011, Photos-Jones and Hall, 2014; Photos-Jones et al., 2017, Photos-Jones et al., 2015, Photos-Jones et al., 2016).

The same issue of confidence in the choice of the appropriate geoarchaeological materials sampled can also be applied to their ecological analysis. The population distributions of microbiota associated with a crude mineral may be considered a biomarker for a particular set of environmental conditions, and can be identified by DNA sequencing. Therefore, the dominant microorganisms identified in the Kean and Lemnian mineral samples should correspond to those which survive well in the original environment. This is particularly true when minerals are toxic or stressful to other bacteria, such that only adapted species can survive. We shall demonstrate that this is particularly true for one Kean sample (730.2, Table 2), meaning that the select Pb toxicity-resistant microorganisms identified today by DNA sequencing correlate well with the high Pb concentration measured in the said sample.

The following sections present methods (Section 2), results (Section 3) and discussion (Section 4). The Appendix gives the full DNA sequencing of the samples' microorganisms.

## 2 Methods

### 2.1 Mineralogy

Four samples (730.2, 730.3, 730.4, and 730.5, Figs. 2a-d) were subjected to a number of analyses. The mineralogical composition of the samples was determined with X-ray diffraction (XRD). The four samples were examined at the School of Mineral Resources Engineering, Technical University of Crete, on a Bruker D8 Advance Diffractometer equipped with a Lynx Eye strip silicon detector, using Ni-filtered CuKα radiation (35 kV, 35 mA). Data were collected in the 2θ range 3–70° 2θ with a step size of 0.02° and counting time 1 s per strip step (total time 63.6 s per step). The XRD traces were analyzed and interpreted with the Diffract Plus software package from Bruker and the Powder Diffraction File (PDF). The quantitative analysis was performed on random powder samples (side loading mounting) emplaced in Al-holders, by the Rietveld method using the BGMN computer program (Autoquan© software package version 2.8).

### 2.2 Geochemistry

Two types of materials were analyzed: solid samples consisting principally of Fe-oxyhydroxides and aqueous solutions here referred to as “crudes” and “leachates” respectively. The latter were produced by adding 0.2 g of the crudes in 5 ml distilled water, dispersing with ultrasonic probe for 20 s, allowing standing for 1 h and subsequent centrifugation. The supernatant was stored in polyethylene bottles for ICP-MS analysis. In the case of the crudes, about 100 mg of each sample were digested in 10 ml concentrated HNO_3_, 2 ml concentrated HCl and 1 ml concentrated HF acids in teflon beakers on a hot plate with addition of 6 ml 0.625% EDTA at boiling point, following the EPA3051 method. The chemical composition of the samples and the leachates was determined by ICP-MS (7500CX coupled with Autosampler Series 3000, both by Agilent Technologies). The method is not suitable for determination of Si due to the use of HF in digestion and S due to analytical constraints. The precision of the analyses was tested using suitable standards. The relative standard deviation of the analyses varied according to the concentration, typically 7% for the major elements, less for the trace elements.

### 2.3 Nanoparticle component

#### 2.3.1 Dynamic Light Scattering (DLS) size and zeta potential data

Particle size for the samples were measured by DLS using a Malvern Instruments Zetasizer nano ZS with a red (366 nm) laser. A method was developed to remove the larger particles, and provide stable suspensions of the smaller particles for DLS analysis. The powders were dispersed in a 0.2% suspension of Novachem surfactant (Postnova Analytics UK Ltd.) in ultrapure water. The suspension was shaken thoroughly, vortexed (Rotamixer, Hook and Tucker Ltd.) at full power for 30 s and treated in the ultrasonic bath (Branson, 1510) for 5 min. The samples were centrifuged at 3000 rpm, for 5 min in 15 ml tubes using an Eppendorf 5804R centrifuge. This removed the larger particles from the samples. Stable suspensions were obtained under these conditions.

The zeta potential measurements of these suspensions were negative, and between −40 and −50 mV, due to the effect of the Novachem surfactant which produced a high surface charge. It follows that the zeta potential was altered and so is not representative of the original material. However, this enabled us to stabilise the suspensions and allowed reproducible size measurements to be made for the smallest particulate size fraction.

#### 2.3.2 TEM-EDAX

Approximately 10 mg of powder was suspended in 10 ml of ultrapure water. The suspension was vortexed for 1 min at full power (Rotamixer Hook and Tucker Ltd.) The sample was processed in the ultrasonic bath for 5 min (Branson 1510 ultrasonic bath) and centrifuged in 15 ml tubes in the Eppendorf centrifuge 5804R at 4000 rpm for 10 min for clay samples, and 2500 rpm for 11 min for iron oxide samples, to remove particles above 450 nm. A drop of 35 μl of the supernatant was deposited onto 200 mesh copper grids with carbon film, and left there without drying for one hour. The excess sample was wicked from the grid, and the grid was washed 4× in water to remove any salts. The grid was dried for 16 h before use. TEM images and EDX measurements were performed at the Birmingham University Centre for Electron Microscopy, using a Jeol JEM 2100 instrument.

### 2.4 DNA sequencing

High-throughput genetic sequencing has emerged as an effective method for determining microbiological composition. It involves the extraction of DNA from the entire community from each sample, and selectively targeting specific genes and amplifying those sequences to facilitate identification of the host microorganism, which involves the 16S-rRNA (small-subunit ribosomal RNA) gene for bacteria and 18S-rRNA (analogous gene) for fungi. Using targeted metagenomics, sequencing operations are capable of identifying over 25 million reads per sample, which is particularly powerful for knowing the organisms' presence in a microbiome.

Samples were aseptically collected on site and maintained under either refrigerated or frozen conditions. At the laboratory, sterile and highly clean conditions were maintained in the handling and transfer of samples, including the use of a biological containment cabinet, which was pre-irradiated with UV and maintained HEPA-filtered laminar air flow, for transferring samples to minimise contamination.

DNA was extracted using MoBio PowerSoil Extraction kits (Qiagen) according to manufacturer's procedures, but sample materials were agitated using a FastPrep24 cell homogeniser (MP Biomedicals; 6.0 speed, 2 × 20 s). Additionally, samples were initially incubated at 70 °C for 10 min to facilitate the DNA extraction from Gram-positive microorganisms. Purity and quantity of extracted DNA were measured using UV-micro-spectrophotometry. Samples were routinely diluted 1:50 with molecular-grade water to minimise inhibitors and improve reaction efficiency of downstream enzymatic processes, and extracts were stored at −80 °C.

Polymerase chain reaction (PCR) was used to selectively target the hypervariable V4 region of the 16S-rRNA gene. Primers consisted of the forward (AYTGGGYDTAAAGNG; position 563–577) and combined set of reverse (TACNVGGGTATCTAATCC, TACCRGGGTHTCTAATCC, TACCAGAGTATCTAATTC, CTACDSRGGTMTCTAATC; position 907–924). To minimise cost, primers were further ‘bar-coded’ with a short 8-base genetic sequence to allow multiple samples to be simultaneously sequenced and sorted post-analytically using RDP initial pipeline bioinformatics tool (Cole et al., 2014; http://pyro.cme.msu.edu/).

Each 100 μl PCR reaction mixture consisted of 10 μl of diluted DNA sample, 50 μl Taq-PCR Master Mix (Qiagen; consisting of 1.5 mM MgCl_2_, 2.5 units of Taq DNA polymerase, 1× proprietary PCR buffer, and 200 μM of each dNTP), 10 μl 10×-primer mixture (0.2 μM final concentration of each primer). Reaction conditions were as follows on a BioRad iCycler5 (BioRad, Hercules, CA USA) instrument: 3 min initial denaturation (94 °C); 30 cycles of: denaturation (30s at 94 °C), primer annealing (30s at 58 °C), and product extension (1 min at 72 °C); and a final extension (10 min at 72 °C). When completed, the instrument maintained the samples at 8 °C. To remove excess primers and un-polymerised nucleotides, PCR products were purified using Qiaquick PCR Purification kit (Qiagen). Quantities of PCR product were quantified by UV micro-spectrophotometrically, combined and condensed to 30 μl, yielding concentrations >20 ng/μl for sequencing.

Library preparation (e.g. adapter ligation) and MiSeq high-throughput sequencing (Illumina) were conducted by GATC-Biotech (Konstanz, Germany) with full quality-control and quality-assurance. The number of MiSeq reads per sample varied. Phylogenetic identity of each sequence was determined based on alignments with the “Classifier” function (Wang et al., 2007) of the RDPpipeline, which maintains databases for 16S- (and 18S-) rRNA sequences (Cole et al., 2014). The bootstrap cutoff was predetermined to be 70% based on sequence length.

### 2.5 Bioactivity-bacterial strains

Reference bacterial strains were used for the antimicrobial testing. The selected species were *Pseudomonas aeruginosa* NCTC 10662 (Gram-negative) and *Staphylococcus aureus* NCTC 12493 (Gram-positive). The choice of the specific strains was based on their common use as environmental indicators, their impact on public health and their relatively simple growth requirements. The desired concentration of bacterial cells in each case was estimated measuring the optical density of the inoculum at 600 nm (Shimadzu UV1240 spectrophotometer). According to the McFarland scale, an absorbance of 0.132 corresponds approximately to a cell density of 1.5 × 10^8^ CFU/ml.

#### 2.5.1 Sample preparation

The antibacterial activity of samples was screened against both bacterial strains using their a) powder (crude) form and b) leachate. Powder samples were obtained mixing each sample with sterile deionised water at a concentration of 600 mg/ml, followed by sterilization at 120 °C for 20 min. All antimicrobial tests with powder samples were performed under continuous stirring of the samples on a magnetic stirrer for homogenization of the used solutions. Aqueous leachates of the samples were prepared at a concentration of 600 mg/ml, mixing samples with sterile deionised water, followed by ultrasonication (Julabo ultrasonic bath) for 30 min at 25 °C and centrifugation at 10000× for 15 min to remove all solids from the solution. The leachate was decanted, sterilised in the autoclave (20 min, 120 °C), and tested against the bacteria.

#### 2.5.2 Antimicrobial tests

Antimicrobial activity of the samples was studied using broth microdilution method and estimating the Minimum Inhibitory Concentration (MIC). MIC is defined as the lowest concentration of an antimicrobial agent that will inhibit the visible growth of a microorganism after overnight incubation. MICs were estimated labelling 96-well sterile microtiter trays with dilutions of each sample. The tested concentration range was 600–0.8 mg/ml. The bacterial inoculum in each case was prepared using LB broth (LABM) and was added in each well after adjustment to 10^5^ CFU/ml. The bioactivity of each sample was estimated in relation to positive control wells, which contained only the bacterial inoculum in LB broth. Microtiter trays were incubated at 37 °C for 18–24 h, followed by optical density measurement at 630 nm, using a microplate reader (Labtech LT-4000 Plate Reader) and Manta LML software.

## 3 Results

### 3.1 Geology–mineralogy (Table 1)

Kea lies within the Cycladic Blueschist belt (Attic Cycladic Crystalline (ACC) and the island's geology is indicative of high pressure/low temperature regional metamorphism (Iglseder et al., 2011; Rice et al., 2012). The dominant rock types are mainly calcareous chloritic schists and marbles. The mineralisation consists of Fe oxides/hydroxides and occurs at the zone of contact between the schists and the marbles or within the marbles of the Blueschist Unit, filling cavities. The presence of precursor pyrite and galena in the Fe-ores and replacement textures of pyrite suggests formation from hypergene alteration (deep weathering) of primary sulfide ore bodies and possibly siderite, suggesting the genesis of gossan. The Fe-Pb mineralisation in Kea has similar genesis and geological and mineralogical characteristics, but on a much smaller scale to that of Pb-Zn-Ag deposits of Laurion, Attica (Skarpelis and Argyraki, 2009).

**Table 1:** XRD analyses of red Kea and Lemnos iron oxide/clay samples. Data for 700.17 and 700.19 from Photos-Jones et al. (2017, Table 1).

Sample	Locality	Goethite	Hematite	Quartz	Calcite	Muscovite/illite	Kaolinite	Alunite
730.2	Petroussa, Kea, *miltos*	82.1	17.9	nd	nd	nd	nd	nd
730.3	Orkos, Kea, yellow ‘*miltos*’	90.3	0.8	2.4	nd	6.5	nd	nd
730.4	Trypospilies, Kea, *miltos*	52	8.1	6.8	33.1	nd	nd	nd
730.5	Orkos, Kea, *miltos*	39.7	7.1	5.9	39.1	6	2.2	nd
700.17	Pharmacy Museum, Basel red Lemnian Earth sample	nd	3.8	17.7	nd	41	37.4	nd
700.19	Kotsinas, Lemnos, red clay sample	nd	1.8	6.4	nd	nd	69.3	22.7

The main Kea ore bodies occur in the Petroussa area in the south, the Orkos area in the east and the Trypospilies area in the north of the island (Fig. 1c). The mineralisation consists mainly of Fe-oxides/oxyhydroxides ± galena, fluorite and barite. It occurs within brecciated marble horizons, which often display dolomitization, filling cavities (e.g. Orkos area). In Petroussa, the ore body occurs in highly brecciated marbles (formation of microbreccia/rock flour) often veined by white calcite, and in stratabound bodies within brecciated carbonate rocks (area between Schoinos Bay and Artelas Bay).

The Kea samples (730.2–730.5) consist mainly of goethite with or without calcite as major constituent and with varying amounts of hematite (Table 1). The Fe-oxyhydroxide/oxide content of the samples is inversely correlated with the presence of calcite. Lemnian Earth (700.17) consists mainly of clay minerals, namely illite and kaolinite and, to a lesser extent, hematite and quartz (Photos-Jones et al., 2017). A red clay sample (700.19) from the locality of extraction of the Lemnian Earth (‘to aima tou Hephaistou’, Kotsinas, NE Lemnos) and presented here for comparison was found to contain, additionally, alunite.

#### 3.1.1 Summary

The Kea (geological) samples consist of iron oxides (with or without calcite) (Table 1); the Lemnian Earth (archaeological) sample 700.17 is a layered alumino-silicate consisting of kaolinite and muscovite/illite. The geological sample of Lemnian clay sediment from Kotsinas (700.19) is rich in alunite. This is corroborating evidence to the information in G-R texts regarding the astringency of Lemian *miltos*. All five samples contain varying amounts of hematite (1–18%). Of the four Kea samples, 730.4 and 730.5 are mineralogically similar. 730.2 is a ‘pure’ iron oxide and 730.3 is mainly iron oxide with 10% quartz and muscovite/illite.

### 3.2 Geochemistry (Table 2)

The chemical compositions of the crude samples and their leachates are listed in Table 2. 730.2 and 730.3 contain large amounts of Fe (~50%) reflecting the abundance of goethite and hematite, whereas 730.4 and 730.5 are rich in Ca (13–15%) reflecting the presence of calcite. Na in the leachate is high for the Orkos (730.3) and the Trypospilies (730.4) samples only. Since Na-bearing phases were not detected in the mineralogical analysis, it is suggested that the high Na content in mica-free samples, might be due to sea-spray adsorbed on the sample surface, although no Cl^−^ ions were measured. Alternatively, in mica-bearing samples, some Na may be hosted in mica. All samples contain Al, Ti, Mn, Mg, As and Zn, and 730.2 is rich in Pb. 730.3, 730.4 and 730.5 have elevated amounts of As, Sb, Zn and Ni. The analyses of the aqueous leachates consist mainly of Ca, Na, Mg, Li and K. There is no Fe in the leachates.

**Table 2:** Results of the ICP-MS analysis (in ppm). C:crude; L:leachate; <DL: below limit of detection.

	730.2C	730.2L	730.3C	730.3L	730.4C	730.4L	730.5C	730.5L	700.17
	Crude	Leachate	Crude	Leachate	Crude	Leachate	Crude	Leachate	Leachate
Li	2.3	0.01	6.9	3.6	7.1	16.7	9.7	0.01	24
B	90.3	0.06	103.4	tr	18.2	0.1	18.6	0.34	16
Na	30,931	0.02	20,640	194.9	23,342	15.4	16,964	0.01	<DL
Mg	1509	54.7	2886	388.3	2339	26.4	2551	21	<DL
Al	8736	11.2	17,348	tr	17,631	15.6	23,415	<DL	<DL
Si	<DL	6	<DL	1.5	<DL	26.6	<DL	7.2	<DL
K	9329	0.01	7563	5.9	7406	14.1	6274	0.01	<DL
Ca	7967	228.9	2623	968.5	132,471	303.8	156,443	306.4	<DL
Ti	230	tr	157.6	<DL	203.9	<DL	449.2	0.01	216
V	34.5	<DL	158.8	<DL	61.9	0.01	81.5	<DL	51
Cr	170.6	<DL	157.3	<DL	103.5	<DL	59.7	<DL	38
Mn	46	tr	5042	0.1	1035	0.83	1014	tr	65
Fe	510,519	tr	499,185	0.02	383,597	0.83	317,906	tr	nm
Co	8	<DL	27.7	0.01	17.6	0.01	16.2	<DL	1
Ni	4.3	0.01	178.5	<DL	89.7	<DL	53.5	0.01	4
Cu	2	<DL	80	<DL	24.3	<DL	40	0.01	17
Zn	475	0.03	1423	0.31	328	0.05	318	0.03	14
As	108	<DL	1037	<DL	795	<DL	502	<DL	77
Se	<DL	<DL	<DL	<DL	<DL	<DL	<DL	<DL	<DL
Rb	8.8	<DL	12.5	0.01	12.8	0.01	15.3	<DL	52
Sr	5.7	0.06	27.6	0.21	17.1	0.06	14.8	0.04	52
Y	<DL	DL	35.7	<DL	12.2	<DL	13.2	<DL	<DL
Mo	15.3	0.01	29.5	0.01	32	0.03	18.8	0.02	<DL
Cd	<DL	<DL	<DL	<DL	<DL	<DL	<DL	<DL	<DL
Sn	61.3	<DL	14.6	<DL	64.6	<DL	72.1	<DL	<DL
Sb	79	tr	1102	<DL	1058	<DL	812	0.01	<DL
Cs	2.81	tr	4.01	0.02	4.43	0.06	11.97	<DL	12
Ba	63	0.02	828	0.05	469	0.04	220	0.02	136
Hg	<DL	<DL	<DL	<DL	<DL	<DL	<DL	<DL	<DL
Pb	5773	<DL	104	<DL	42	<DL	64	<DL	10
U	<DL	<DL	<DL	<DL	<DL	<DL	<DL	<DL	<DL

#### 3.2.1 Summary geochemistry (powders) (Table 2)

730.2 is rich in Pb (*c*. 6000 ppm) and the remaining Kea samples have elevated concentrations of metallic (toxic) elements (As, Cr, Zn, Ni); the Lemnian Earth (730.17) has elevated Ti, Ba and Mn. As regards the leachates, the compositions (with the exception of light elements) of the Kea samples indicate an absence of toxic elements.

### 3.3 Nanoparticle component: DLS size and TEM/EDAX (Table 3, Table 4)

Table 3 presents the nanoparticle size distribution. The average particle sizes of the Kea iron oxides were slightly smaller than the Lemnos sample (200.5 ± 3.0 nm). This is probably due to the effect of centrifugation on a higher density material. The red *miltos* (730.4) was the finest with an average particle size of 168.9 ± 2.6 nm. It was originally thought that ‘yellow *miltos*’ (730.3) may have had a larger particle size range but this was not the case (see reference to Theophrastus' comment about yellow ‘ochre’ in the Introduction and the Discussion).

**Table 3:** DLS size results: *Z*-average size data with standard deviation. Both yellow ochre (730.3) from Orkos and *miltos* (730.4) from Trypospilies have comparable naturally fine particle sizes. Lemnos samples (700.17 and 700.19) are slightly coarser.

	Name	Material	Average diameter	SD	Minimum size (nm)	Maximum size (nm)
730.3	Yellow ochre -Orkos, Kea (geological sample)	Iron oxides	177.9	0.9	50	459
730.4	*Miltos* red- Trypospilies, Kea (geological sample)	Iron oxides	168.9	1.3	33	531
730.17	Red Lemnian Earth/*miltos*, Basel, Switzerland (archaeological sample)	Red ‘clay’	200.5	1.5	59	700
730.19	Red clay, ‘*aima tou Hephaistou*’, Kotsinas, Lemnos (geological sample)	Red ‘clay’	209.9	1.7	68	615

**Table 4:** TEM-EDX analysis of nano-scale sub-samples in Figs. 3–6.

Weight %	O	Al	Si	Fe	Ti	Co	P	S	K	Mn	Mg	Au	Ta	V	Sr	Ba	Ca	Ni
730.3a	38.13	0.74	1.06	59.32	nd	nd	0.24	nd	nd	0.26	0.24	nd	nd	nd	nd	nd	nd	nd
730.3b	40.67	0.36	0.69	57.73	nd	nd	nd	nd	nd	nd	nd	0.56	nd	nd	nd	nd	nd	nd
730.3c	60.66	nd	13.31	26.03	nd	nd	nd	nd	nd	nd	nd	nd	nd	nd	nd	nd	nd	nd
730.4a	43.98	1	2.56	52.46	nd	nd	nd	nd	nd	nd	nd	nd	nd	nd	nd	nd	nd	nd
730.4b	44.56	0.48	7.73	47.23	nd	nd	nd	nd	nd	nd	nd	nd	nd	nd	nd	nd	nd	nd
730.4c	32.62	1.51	nd	16.82	nd	nd	nd	nd	nd	nd	nd	nd	49.05	nd	nd	nd	nd	nd
700.17a	33.93	0.91	2.85	2.52	57.13	2.42	nd	nd	nd	nd	nd	nd	nd	0.25	nd	nd	nd	nd
700.17b	52.91	1.27	38.75	6.75	0.12	nd	nd	nd	0.19	nd	nd	nd	nd	nd	nd	nd	nd	nd
700.17c	35.96	0.25	0.37	2.38	59.82	0.31	nd	nd	nd	nd	nd	0.46	nd	0.45	nd	nd	nd	nd
700.19a	47.33	6.76	25.67	11.41	0.13	nd	1.08	4.49	1.69	nd	nd	nd	nd	nd	0.3	0.32	0.41	nd
700.19b	53.44	7.25	26.3	9.78	0.32	0.34	0.37	1.37	0.62	nd	nd	nd	nd	nd	nd	nd	0.2	nd
700.19c	48.1	13.36	9.43	12.31	1.03	0.72	nd	10.23	4.55	nd	nd	nd	nd	nd	nd	nd	nd	0.29

nd = not determined.

**Figs. 3–6:**
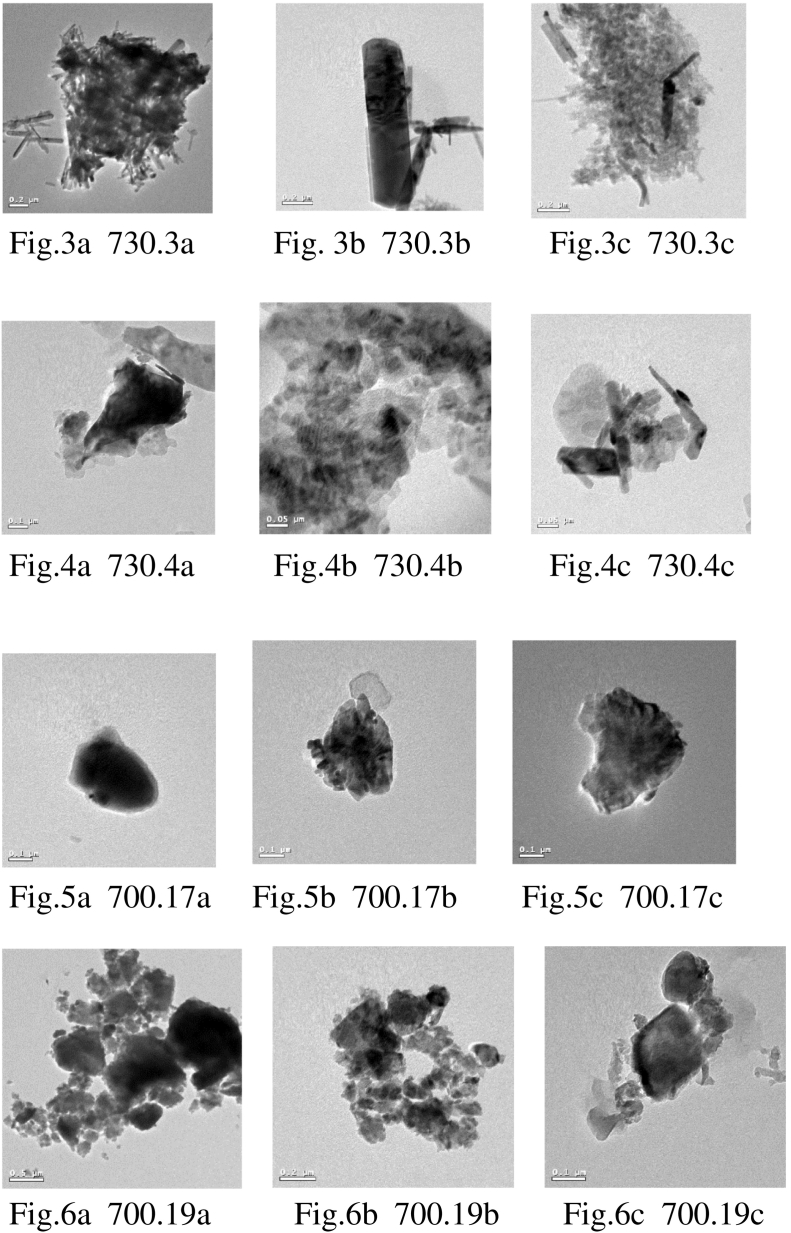
TEM images of 730.3 and 703.4 (from Kea) and 700.17 and 700.19 (from Lemnos).

TEM–EDX analysis (Table 4), although semi-quantitative with accuracy at about the percent level, is useful where the material is heterogeneous, as it allows the variation between particles to be investigated. It may be possible to assign different compositions to different physical forms observed under the electron microscope. Figs. 3–6 should be examined in association with Table 4. H, C and Cu are excluded from the analysis. Each image displays a single particle in the suspension which may contain many smaller crystallites; these are bound together with water or possibly organic material when they were deposited. The EDX analysis gives an average composition of the particle.

#### 3.3.1 730.3 and 730.4

Individual nano-sized particles can be seen in Figs. 3 and 4. They have similar compositions, and appear to be predominately iron oxide/hydroxide (Figs. 3a,b, 4a,b and Table 4). This is consistent with the XRD data in Table 1 which shows high levels of goethite, FeO(OH). These particles also contain around 2% of clay minerals, and 730.3c has a higher proportion of silica than is usually found in clays, so silica may also be present. Silica is present in the XRD of the bulk material in the form of quartz. Note that 730.3 and 730.4 are free of clay minerals (Table 1). This apparent inconsistency is due to the presence of clay-size illite particles in 730.3 (Table 1) and/or the presence of clay minerals in abundances below the detection limit of the XRD technique. Since clay minerals are below 2 μm in size they are enriched in the nanoparticles fraction. The images of the nano-subsamples 730.3a–c and 730.4a–c show clear differences between the two materials, despite their similarity in composition. 730.3a–c contain needle-shaped crystals, whereas 730.4a–c appear to be made up of more plate-like crystals.

Nano-sized particle 730.3a also contains low levels of Mn, Mg and P, and 730.3b is shown to contain a low level of Au. 730.3c (Fig. 3c) has a different composition from those discussed above. No aluminium was detected in this sample, but the composition analyzed is consistent with equal quantities of silica and iron oxide/hydroxide. Both these materials are in evidence in the bulk XRD analysis as quartz and goethite. In complete contrast to 730.3a–c and 730.4a–b, 730.4c (Fig. 4c) has almost equal atomic ratios of iron and tantalum. Aluminium and oxygen are also present but not in clays, as there is no silica present. The XRD of 730.4 shows a high level of calcium carbonate in the form of calcite, but no calcium was found in the particles examined by EDX, suggesting that this does not make up a high proportion of the nanofraction.

#### 3.3.2 700.17

Nano-sized particles 700.17a and 700.17c (Fig. 5a and c, respectively) in the archaeological sample of Lemnian Earth were very similar in composition. The atomic ratios suggest the particles are mainly TiO_2_, although the oxygen levels recorded are both slightly lower than expected at an atomic ratio of 1.8, rather than 2. Other metals present are Fe (2.5%), V (0.25%) and Co (0.3–3%). Clay minerals are also present (<5%). The XRD data shows illite, kaolinite and quartz as the main phases in the bulk sample of 700.17 which accounts for the clay minerals present, but does not show any phases containing titanium. The EDX data would suggest that titanium-containing particles are more prevalent in the small particle fraction rather than in the bulk material. The composition of 700.17b is much higher in silica with low levels of Fe, Ti, Al and K present, reflecting the large percentage of quartz.

#### 3.3.3 700.19

Nano-sized particles 700.19a–c (Fig. 6a–c) in the red clay from Kotsinas show polycrystalline clusters. 700.19a and 700.19b have much higher levels of Si than 700.19c. The presence of S (1–10%) and K (0.5–5%) in all three particles, and P in two suggest that sulphate (alunite) and phosphate (unknown) minerals are present. 700.19a-c have a much wider range of metals present than the other samples examined here. 700.19a contains low levels of Sr, Ba and Au, and Ca is present at low levels in both 700.19a and 700.19b. Co is found in 700.19b and c, and Ni is recorded in 700.19c.

#### 3.3.4 Summary nanoparticle component (Table 3, Table 4)

Average nanoparticle size for the Kea samples particles is slightly smaller (*c*. 170 nm) than for the Lemnian Earth sample (200 nm); the nanoparticle mineralogy of the Kea samples consists of iron oxides and clay particles; for the Lemnian Earth it consists of clay particles and titanium oxide. Tantalum oxide is present in the leachate of 730.4. There is no difference in the particle size between yellow and red *miltos*. It should be noted that, although very little Fe is measured in the ICP leachates, the nanoparticle component is Fe-rich. This is probably because Fe oxide is very insoluble, and so it would not be present in the leachate; instead it will primarily be concentrated in the nanoparticulate fraction.

### 3.4 The microbial ecology of Kea mines (Table 5)

We characterised the microorganisms in the samples using extracted DNA and 16S-rRNA targeted high-throughput sequencing. The samples had relatively little DNA (<0.5 μg-extracted DNA/g soil or <1 ng/μl in the DNA extract), and preliminary DNA screening suggested most were bacterial in origin; however, trace signatures of fungal and chloroplast (plant) DNA were found in 730.2 and 730.3. The overwhelming abundance of bacterial DNA has led us to focus on this aspect of the microbiological community. Detailed characterisation of microbial species associated with Kea samples is presented in the Appendix and relative distributions are shown in Table 5.

**Table 5:**
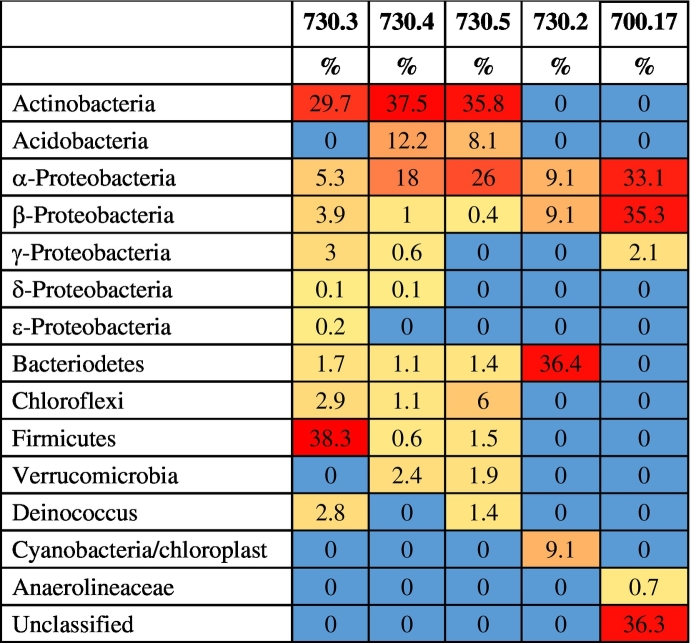
Heat map for relative distribution of microorganisms amongst five samples, based on DNA sequencing. 0% represents amounts 0.1–0.5%. For Table of results, see Appendix.

Most immediately, there is a visible difference between 730.2 and the remaining three since the pattern of DNA concentrations reflects the toxicity of the local environment from which these samples derive. For example, 0.5% Pb in the Petroussa sample (730.2) would be sufficiently toxic to bacteria and possibly has contributed to the minimal phylogenetic reads (n < 200). On the other hand, the remaining samples (730.3, 730.4 and 730.5) had sufficient DNA for phylogenetic characterisation despite the presence of toxic trace elements like As and Zn (Table 2), suggesting that either the elements had low bio-availabilities or the bacteria were resistant.

The relative distribution of bacterial phyla associated with each of the samples (Table 5) further highlights an apparent disparity amongst the samples. Whereas 730.2 shared very little resemblance with the other samples (it had signatures for unclassified Flavobacteriales, *Sulfurimonas*, *Alkanindiges*, and a chloroplast), 730.4 and 730.5 had similar relative distributions to each other, and closely resembled 730.3 in terms of dominance of Actinobacteria. In terms of any functional roles related to the microbial community in Petroussa (730.2), it remains rather difficult to draw any major conclusions given the limited DNA-sample size. *Flavobacterium* spp. are ubiquitous in soil and freshwater bacteria; *Sulfurimonas* spp. are autotrophic, denitrifying bacteria, capable of obtaining energy from sulfur and hydrogen; and *Alkanindiges* spp. are associated with hydrocarbon degradation. The other three samples had high representations of Actinobacteria and α-proteobacteria, particularly in terms of actinomycete and rhizobial bacteria, respectively, suggesting common soil-related bacteria (Table 5). Actinomycetes can represent 10–30% of soil bacteria (Atlas and Bartha, 1998); they resist desiccation and have preference for neutral to slightly alkaline conditions.

Many bacteria are recognised for their importance for plant health, e.g. many of the Fimicute bacteria (especially in 730.3: *Paenibacillus*, *Ammoniphilus*, *Microvirgus*), α-proteobacteria (Rhizobiales; ranging from 3 to 6% relative abundance), and Frankineae (a group of Actinomycetes; 2–6%). More specifically, *Paenibacillus* is recognised as a growth promoter for plants (Bloemberg and Lugtenberg, 2001; Kloepper et al., 1980) via phosphate solubilisation, nitrogen fixation and disease control (antibiotics). Although *Frankia* spp. were not specifically recognised, similar genera from the suborder Frankineae were found associated with soils, many of which are highly tolerant of extreme (barren) conditions, for example, *Blastococcus*, *Geodermatophilus* and *Modestobacter*. Many nitrogen-fixing microorganisms (e.g. within Rhizobiales and Frankiaea clades), not only add fertility to barren soils, but also excrete porphyrins which act as metal (M^+2^) chelators and antibiotic precursors. These compounds have been used to coordinate activities in nanoparticles (Gu et al., 2005).

Often associated with plant-growth promotion and the sequestration of heavy metals (including iron) are the Burkholderiales bacteria. *Burkholderia* spp. have been previously characterised along with the similar *Pseudomonas* spp., which have been extensively studied for their siderophore production (Venturi et al., 1995). Often reputed for their membership of animal and plant pathogens, Burkholderiales and many Rhyzobiales have also been found to be associated with plant health related to heavy metal resistance and sequestration (e.g., Nocelli et al., 2016), phosphate solubilisation (Rodrigues and Fraga, 1999) and secretion of growth factors (Vessey, 2003).

There were also bacteria commonly associated with sulfur and bacterial photosynthesis. They represent the autotrophic bacteria capable of fixing their own carbon via light exposure: Rhodobactereae and Rhodospirillaceae. They were relatively abundant in 730.4 and 730.5 (3–6% of total bacteria population), but less in 730.3 (<1%). *Rhodobacter*, in particular, has been reported to facilitate the oxidation of ferrous iron (Ehrenreich and Widdel, 1994). Deinococcus phylum of bacteria, *Rubobacter*, *Solirubrobacter* and *Breveundimonas*, have the reputation of being capable of surviving harsh environments, which can reflect the landscapes of the region.

Finally, there are guilds of bacteria representative of possible contaminants. The samples were originally collected without any consideration for future microbiological assays; as a result, inadvertent contamination by handling cannot be excluded (though they may also be present in soils). Examples of foreign bacteria include species of *Streptococcus*, *Propionobacterium*, *Staphylococcus*, Neisseriales, Pasteurellaceae, *Dietzia*, *Mycobacteria* and *Corynebacteria*. Fortunately, relative abundances were <1%.

In reference to 700.17 (Lemnian earth/miltos), the sample contained Rhizobiales and Bradyrhizobium bacteria (α-proteobacteria), bacteria commonly associated with nitrogen-fixation and porphyrin excretion. *Achromobacter* spp. and *Comamonadas* spp. are known for their ability to degrade hydrocarbons, and commonly found in soils and water (marine and freshwater). Anaerolineaceae represent thermophilic (heat-tolerant) bacteria. *Moraxellaceae and Pasteurellales* represent clinically relevant bacteria found in respiratory tracts - likely to be trace contamination.

#### 3.4.1 Summary microbial ecology (Table 5)

730.2 has a different ecological makeup compared to the other three relatively similar Kea samples: 730.3, 730.4 and 730.5. In particular, 730.4 and 730.5 appear mineralogically and geochemically similar but also with respect to their microbiota. There are differences in (a) the genus-composition of α- and β-proteobacteria and (b) the presence of δ- and γ-proteobacteria in 730.4 but not in 730.5. The microbial ecology of the Lemnian earth/miltos sample is very different.

### 3.5 Bioactivity (Table 6)

The antimicrobial activity of the samples was assessed estimating the MIC values for the powder (crude) samples (Kea samples only) and the leachates (all six samples) (Table 6). The MIC reveals the bacteriostatic rather than bactericidal nature of the samples, indicating inhibition of microbial growth. Regarding leachates, MICs against *P. aeruginosa* ranged from 100 to 300 mg/ml with 730.2 and 730.4 being the most effective for the inactivation of this pathogen. By contrast, lower leachate concentrations (50–150 mg/ml) were required for inhibiting the growth of *S. aureus*, which exhibited higher sensitivity levels. Amongst the samples, the leachate of 730.4 showed the highest antimicrobial activity against both bacteria tested. Table 6 shows the relative bioactivities of the Kea and Lemnos samples for leachates only. The most bioactive sample against both *P. aeruginosa* and *S. aureus* is the Lemnian Earth (700.17).

**Table 6:** Minimum Inhibitory Concentration (MIC) of the tested samples over *P. aeruginosa* and *S. aureus* growth (nd = not determined due to sample unavailability).

Bacterial indicator	MIC (mg/ml)				
	730.2 - leachate	730.3 - leachate	730.4 - leachate	730.5 - leachate	700.17 – leachate
*P. aeruginosa*	100	300	100	300	50
*S. aureus*	75	75	50	150	12.5

	730.2 - powder	730.3 - powder	730.4 - powder	730.5 - powder	700.17 - powder
*P. aeruginosa*	25	40	6.25	12.5	Nd
*S. aureus*	25	40	3.12	50	Nd

Regarding powders (crude) solutions, the results show that these behaved differently on a sample by sample basis, with all powder forms exhibiting considerably higher antibacterial activity by comparison with their leachate form. MIC values of the powder forms ranged between 6.25 and 40 mg/ml and 3.12–50 mg/ml over *P. aeruginosa* and *S. aureus*, respectively. The greatest difference between the powder and the leachate forms was observed with 730.5, which was more effective than its leachate against *P. aeruginosa* and *S. aureus* by a factor of 24 and 3, respectively. 730.3 revealed the lowest difference levels, while the powder form of 730.4 was recorded as the one with the most effective antibacterial properties.

The results verify the fact that the resistance precedence order between Gram-positive (*S. aureus*) and Gram-negative (*P. aeruginosa*) bacteria is not always the same. Comparable results were obtained in the study of Nor et al. (2016), who tested the toxicity of iron oxides over *E. coli* (Gram-negative) and *Staphylococcus epidermidis* (Gram-positive). Comparing the resistance levels of those two bacterial species, the MBC (minimum bactericidal composition) indicated that *E. coli* was more tolerant than *S. epidermidis* under the specific experimental conditions.

The mineralogy and geochemistry of each sample seem to play a role. The main components of the samples, i.e. goethite and hematite, are iron-containing nanoparticles, which are usually non-toxic for microorganisms (Borcherding et al., 2014). However, hematite has been reported as a bactericidal agent in aqueous solutions. Gram-positive bacteria exhibit higher sensitivity than Gram-negative ones in the presence of metal oxide nanoparticles like hematite nanoparticles (Azam et al., 2012). This is in agreement with the current results; 730.2 and 730.4, which contain hematite, showed considerable antibacterial activity with *P. aeruginosa* being more resistant than *S. aureus*. Nevertheless, given that the Fe dissolution from hematite and its overall bioleaching behaviour are considered inadequate for satisfactory microbial elimination (Nor et al., 2016), it is either the concentration of trace elements in solution that affects the antimicrobial properties of the four *miltos* samples and/or the presence/role of the microbiota and their biomolecules.

#### 3.5.1 Summary antibacterial activity (Table 6)

The Kea powders display greater antibacterial activity than the Kea leachates (by one to two orders of magnitude). This may be due on account of their concentration. Amongst the former, 730.4 is the most effective (less than one order of magnitude) followed by 730.5 and 730.2. 730.3 is the least effective. Amongst the leachates and including the Lemnian Earth, the latter is the most effective. Overall the Kea leachates show minimal antibacterial activity. As can be seen from Table 2, heavy metals, although at high concentrations in the powder are negligible in the leachate. Similarly for the Lemnos sample. However Ti as TiO_2_ is present in the latter.

## 4 Discussion

This paper has outlined our multidisciplinary method set in place in previous publications and expanded here to address the nature, properties and applications of Greco-Roman (G-R) minerals as lithotherapeutics (Photos-Jones and Hall, 2011, 2014; Photos-Jones et al., 2017, 2015, 2016). Our approach has been to examine these minerals ‘at source’, the very same geographical areas highlighted by the texts, albeit often vague as to specific localities, to sample and analyse them chemically and mineralogically; more recently we have begun to view them as the products of the interaction between environment and biosphere and therefore, intrinsically, as both organic and inorganic in nature. The question addressed in this paper is: were Kean and Lemnian *miltos* mineralogically, geochemically and eco-microbiologically the same? Which one of these attributes was instrumental in underpinning each of *miltos'* applications?

The five samples examined - four geological and one archaeological - all derive from areas with known mineral extractive activity in antiquity (Kea, various mines 5th–4th c BC; and Lemnos, Kotsinas in the NE of the island). We have not had the opportunity to work in Cappadocia and therefore Sinopic *miltos*, presently remains unknown. We examined each sample in order to assess which of the five would qualify as a good antibacterial or antifouling agent or as a fertilizer. *Miltos*, the ingredient in the medical recipe, the fertilizer or the antifouling agent, was not identical to *miltos* deriving from the Kean mine or the Lemnian clay sediment. Epigraphic evidence as well as the G-R medical and scientific authors are frequently at pains to separate the two but their efforts are often ‘lost in the translation’.

### 4.1 Miltos as an antifouling agent

The Athenian inscription of *c*. 360 BC decreed that Kea *miltos* was exported uniquely to Athens (see note 2). The frequent reference of *miltos* in association with boats suggested not mere decoration but, crucially, boat maintenance as well (Lytle, 2013) or *miltos* as an antifouling agent, i.e. substances that stop the growth of bacterial films on which macroorganisms (e.g. barnacles) can attach themselves. Sample 730.2 is exceptionally high in Pb. As a result, there is a relatively small number of ecological microorganisms associated with it (Table 5 and Appendix) to include bacteriodetes or cyanobacteria/chloroplasts. It follows that, if this sample of *miltos* mixed in an organic medium were to be applied to a wooden surface like a ship's hull, it would not have allowed the growth of biofilm and as such it would have indeed acted as an efficient antifouling agent.

*Miltos* of the 730.3 specification with higher Zn, As and Cu concentrations compared to other Kea samples, and mixed with pitch could also be an effective biofouling agent (the *miltopissa* of the 4th century BC inscription (see note 1)). Antifouling boat paints may contain many biocidal substances, with primary active ingredients being copper and/or zinc, or more likely today, organic biocides (Soroldoni et al., 2017). Various aquatic organisms, such as the macroalgae *Ceramium tenuicorne* and the crustacean *Nitocra spinipes*, have exhibited considerable sensitivity to copper contained in copper-based biocides (Bighiu et al., 2017). Bacteria-generated biofouling is hugely detrimental to the shipping industry even today, and they would have been exceptionally so in antiquity since they considerably reduce speed of movement at sea (e.g. Schultz, 2007).

### 4.2 Miltos in agriculture

A late 4th century BC inscription and ancient texts (see note 3) state that *miltos* - locality unspecified – should be used in agriculture and in association with pitch; *miltos* could also be applied to the roots of trees. In the first instance, the application would be to prevent disease, in the second as a fertilizer. In the first case the medium would be an organic substance, in the latter an aqueous solution.

We examine the use of *miltos* as fertilizer and the contribution of the microorganisms might bring to its bioactivity. In scrutinising the ecological microorganisms associated with 730.3, 730.4 and 730.5, it can be demonstrated that many bacteria identified in the samples are linked with nitrogen-fixation in the soils, and iron is a major component of the enzymes and organic compounds associated with that process. Ultimately, they contribute to sequestration and control of exposure to metals and to the nutritional quality of the mineral matrix; as such, Kean *miltos* of the above specification would have made good ‘fertilisers’, probably applied in a concentrated aqueous solution or as powder at the tree roots.

### 4.3 Miltos as an antibacterial agent (Table 6)

Antibacterial action was understood by the ancient world, but only indirectly. In assessing the response of the five samples to known pathogens, the samples were compared in two forms, as powders and as leachates.

In comparing leachates, the Lemnian *miltos* is the most antibacterial. Kean *miltos* was more antibacterial in powder form than in solution (as a leachate). Metallic elements do not dissolve in aqueous solutions (see Table 2) and so their antibacterial effect is limited. We suggest that the presence of TiO_2_ at the nanofraction level may be responsible for the enhanced bioactivity of Lemnian *miltos*. TiO_2_ is a known antibacterial (Jesline et al., 2015).

We proceed to compare the Kea powders. 730.4 is the most antibacterial of the four powder samples. We would have expected 730.2 to be the most antibacterial given the high Pb concentration in the sample compared to the others. This is not the case and this is perhaps because Pb is toxic, meaning its effect is dose dependent. Toxicity is determined by lethal dose (LD) while antibacterial action is determined by Minimum Inhibitory or Bacteriocidal Concentration.

In order to test the possible contribution of the organic component (microorganisms and their secondary metabolites) of a G-R mineral to antibacterial action it is essential to compare two samples which are very similar chemically, mineralogically and eco-microbiologically. 730.4 and 730.5 are sufficiently similar on all three parameters to allow direct comparisons. The two samples are also similar at the nano-fraction level, but with the exception of Tantalum which is present in the nanofraction of 730.4 but not in that of 730.5. As mentioned, 730.4 is more antibacterial than 730.5 and moreover the antibacterial action towards Gram-positive and Gram-negative pathogens is reversed - i.e. 730.4 is more effective against *S. aureus* than 730.5. However, tantalum does not demonstrate any intrinsic antibacterial activity or ability to inhibit biofilm formation (Harrison et al., 2017), and so we suggest that the difference in antibacterial action may be due to the nature of the ecological microorganisms present in each of the two samples.

Table 5 highlights the differences in ecological microorganisms between the two samples. 730.5 lacks δ- and γ-proteobacteria compared to 730.4, although the total amounts in the latter are very small (*c*. 1.5%). Furthermore, there is only partial overlap at the genus level and within the larger families, for example in α-proteobacteria or other groups such as actinobacteria, and acidobacteria. On this basis, we conclude that it might be the difference at the genus/species level which may have been responsible for a stronger antibacterial action on the part of sample 730.4. Identification of bacterial isolates at a species level requires lengthier DNA sequences, and was not possible within this stage of the investigation. Further work is required to confirm the basis of difference in antibacterial action between these two similar (mineralogically/geochemically) samples; also similar at the phyllum/class level of microbial ecology. This is because different bacteria at the species level can produce extremely different secondary metabolites which may be responsible for the difference in MIC values.

To conclude, we suggest that bioactivity of *miltos*, its effect on living organisms (biofilms, plants or humans) was and is the result of a complex network of interactions between mineral and its microbiome, the organic and the inorganic component contributing, more or less, to the total bioactivity of the *miltos* sample. The G-R world clearly experimented with the natural material, not as a single mineral but as the output of a specific locality (in Kea, Lemnos, or Cappadocia), working at enhancing the properties of the original, with varying results. In the process, it acquired an empirical but thorough understanding of the strengths and limitations of the *miltos* of that specific locality. Unravelling this complex network of empirical knowledge and translating it into the language of physico-chemical and biological sciences will be a challenging but also a thoroughly exciting task.

## Figures and Tables

**Table:** Table of extractable DNA from each sample, based on 16S-rRNA phylogenetic characterisation.

Phylum	Class/Order	Genus
730.2 Petroussa		
Bacteroidetes	Flavobacteriales (36.4%)	*Flavobacterium*
ε−Proteobacteria	Campylobacteriales (9.1%)	*Sulfurimonas*
γ−Proteobacteria	Pseudomonadales (9.1%)	*Alkanindiges*
Chloroplast group	Chloroplast group (9.1%)	Group XIII
Unclassified	Unclassified bacteria (36.3%)	

730.3 Orkos N Gallery		
Firmicutes	Bacillales (34.9%)	*Sporolactobacillus*, *Paenisporosarcina*, *Ammoniphilus*, *Paenibacillus*, *Streptococcus*, *Staphylococcus*
	Clostridia (2.3%)	*Anaerococcus*, *Peptostreptococcus*
Actinobacteria	Solirubrobacterales (2.4%)	*Solirubrobacter*
	Rubrobacterales (2.0%)	*Rubrobacter*
	Pseudonocardinales (11.9%)	
	Actinomycetales- Frankineae (5.9%)	*Blastococcus*, *Modestobacter*
	Propionobacteriales (1.5%)	
	Nocardiodales (1.4%)	*Nocardioides*, *Marmaricola*, *Flindersiella*
	Actinomycetales-Corynebactericeae (0.4%)	*Corynebacteria*, *Trucella*
	Actinomycetales-Streptomycetaceae (0.1%)	*Streptacidiphilus*
	Acidimicrobiia (1.9%)	*Iamia*
α−Proteobacteria	Rhizobiales (2.2%)	*Aminobacter*, *Microvirga*, *Afipia*, etc.
	Sphinomonadales (1.0%)	*Rhizorhabdus*, *Spinosinicella*
	Caulobacterales (1.1%)	*Brevundimonas*
	Rhodospirillales (0.2%)	*Skermanella*
	Rhodobacterales (0.2%)	*Rhodobacterium*
β−Proteobacteria	Burkholderiales (3.7%)	*Telluria*, *Pelomonas*, *Noviherbaspirillium*, *Bordatella*
	Neisseriales (0.2%)	*Morococcus*
γ−Proteobacteria	Pastuerellales (1.0%)	
	Enterobacteriales (1.0%)	*Leclercia*
	Chromatiales (0.7%)	*Thiohalomonas*
	Pseuodmonadales (0.1%)	*Alkanindiges*
	Xanthomonadales (0.3%)	*Xanthomonas*
δ−Proteobacteria	Myxococcales (0.1%)	*Labilithrix*
ε−Proteobacteria	Campylobacterales (0.2%)	*Campylobacteria*, *Sulfuricurvum*
Chloroplast group	Chloroplast group (0.6%)	
Deinococcus	Deinococcales (2.8%)	*Truepera*
Bacteroidetes	Flavobacteriales (1.7%)	*Flavobacterium*
Chloroflexi	Thermomicrobia (1.3%)	*Nitrolancea*
	Anaerolineales (0.1%)	*Anaerolinea*

730.4 Trypospilies		
Actinobacteria	Actinomycetales-Propionobacterinaea (4.7%)	*Nocardioides*, *Kribbella*
	Actinomycetales-Microoccineae (3.5%)	*Arthobacter*, *Janibacter*, *Cellulomonas*
	Actinomycetales-Frankineae (1.6%)	*Blastococcus*, *Geodermatophilus*, *Modestobacter*
	Pseudonocardineae (0.7%)	*Pseudonocardia*, *Saccharothrix*, *Lentzea*
	Actinomycetales-Corynebactericeae (0.2%)	*Dietzia*
	Actinomycetales-Streptomycetaceae (0.1%)	*Streptacidiphilus*, *Streptomycetes*
	Solirubrobacteriales (9.1%)	*Solirubrobacter*, *Conexibacter*
	Rubrobacter (2.1%)	*Rubrobacter*
	Gaiellales (2.7%)	*Gaiella*
	Acidimicrobiaceae (2.3%)	*Aciditerrimonas*, *Ilumatobacter*
Acidobacteria	Various groups (12.2%)	
α−Proteobacteria	Rhizobiales (4.8%)	*Bradyrhizobium*, *Microvirga*, *Mesorhizobium*, *Chelatococcus*, etc.
	Rhodospirillales (3.1%)	*Skermanella*, *Roseomonas*
	Sphingomonadales (1.7%)	*Sphingomonas*
	Rhodobacteriales (1.3%)	*Rubellimicrobium*
	Caulobacterales (1.0%)	*Phylobacterium*, *Brevundimonas*, *Caulobacter*
β−Proteobacteria	Burkholderiales (0.6%)	*Massilia*, *Azohydromonas*, *Achromobacter*, *Thiomonas*, *Lautropia*
	Unclassified β-proteobacteria (0.3%)	
γ−Proteobacteria	Pseudomonadaceae (0.2%)	*Pseudomonas*, *Acinetobacter*
	Enterobacteriaceae (0.1%)	*Enterobacter*
	Unclassified γ-proteobacteria (0.3%)	
δ−Proteobacteria	Desulfovibrionales (<0.1%)	*Desulfovibrio*
	Bdellovibrionalles (<0.1%)	*Bdellovibrio*
	Desulfobacteriales (<0.1%)	*Desulfobacter*
	Syntrophobacteriales (<0.1%)	
	Myxococcales (<0.1%)	
Verrucomicrobia	Spartobacteria (1.9%)	
	Unclassified Verrucomicrobia (0.3%)	
Chloroflexi	Thermomicrobia (0.3%)	*Sphaerobacteria*
	Caldilineae (0.1%)	*Litorilinea*
	Unclassified Chloroflexi (0.7%)	
Bacteroidetes	Various (1.1% total; <0.1% each)	
Firmicutes	Various (0.6% total; <0.1% each)	

730.5 Orkos N Gallery		
Actinobacteria	Actinomycetales-Frankinea (5.8%)	*Blastococcus*, *Modestobacter*, *Geodermatophilus*
	Actinomycetales-Micrococcineae (4.9%)	*Renibacterium*, etc.
	Actinomycetales-Propionibactereae (4.4%)	*Marmoricola*, *Nocardioides*
	Actinomycetales-Streptomycinea (1.5%)	Various
	Unclassified Pseudonocardineae (0.9%)	
	Actinomycetales-Micromonosporineae (0.3%)	*Polymorphospora*
	Actinomycetales-Cornyebacterineae (0.1%)	
	Solirubrobacteriales (7.6%)	*Solirubrobacter*, *Conexibacter*
	Rubrobacteriales (1.6%)	*Rubrobacter*
	Acidimicrobiaceae (3.1%)	*Aciditerrimonas*, *Ilumatobacter*, *Iamia*
	Gaiellales (2.7%)	*Gaiella*
	Thermoleophilia (1.3%)	*Thermoleophilum*
α−Proteobacteria	Rhodospirillales (6.2%)	*Skermanella*, *Acetobacter*, *Rhodospirillum*
	Rhizobiales (5.6%)	*Microvirga*, *Salinarimonas*, *Methylorosula*
	Caulobacterales (3.8%)	*Woodsholea*, *Phenylobacterium*
	Sphingomonadales (3.4%)	*Sphingosinicella*, *Rhizorhabdus*, *Stakelama*
	Rhodobacterales (3.1%)	*Rubellimicrobium*, *Rhodobacter*
β−Proteobacteria	Burkholderiales (0.4%)	*Oxalicibacterium* etc.
	Unclassified bacteria (0.4%)	
Acidobacteria	Various groups (8.1%)	
Chloroflexi	Thermomicrobia (4.6%)	*Nitrolancea*
	Caldinlineae (0.3)	*Litorilinea*
	Unclassified Chloroflexi (1.1%)	
Verrucomicrobia	Spartobacteria (0.8%)	*Terrimicrobium*
	Unclassified Verrucomicrobia (1.1%)	
Firmicutes	Bacillales (1.2%)	*Planomicrobium* etc.
	Unclassified Firmicutes (0.3%)	
Bacteroidetes	Cytophagia (0.9%)	*Adhaeribacter*, *Rhodocytophagia*
	Sphingobacteriales (0.5%)	*Segetibacter*
Deinococcus	Deinococci (1.4%)	*Deinococcus*, *Truepera*

700.17		
α−Proteobacteria	Rhizobiales (33.1%)	*Bradyrhizobium*, *Rhizobium*
β−Proteobacteria	Burkholderiales (35.3%)	*Achromobacter*, unclassified *Comomonadaceae*
γ−Proteobacteria	Pseudomonadaceae (2.1%)	*Alkanindiges*
	Unclassified Pasteurellales (2.1%)	
Chloroflexi	Anaerolineales (0.7%)	*Anaerolinea*
Unclassified	Unclassified bacteria (32.8%)	

## Footnotes

1 As a cosmetic miltos appears in Herodotus (*Hist*. 4.191; 7.69) in reference to body paint amongst African (Libyan and Ethiopian) tribes. Actors used it (Athenaeus 5.197f–198a), as did Aristophanes himself who appears to have made use of it instead of a mask. In 4th century BC Athens, women normally applied red plant-based cosmetics (ἔγχουσα) rather than (red) minerals, but it appears that also some men did opt for *miltos*, dispersed in olive oil, to rub their bodies with, a practice rather frowned upon by Xenophon (*Oeconomicus* 10). Hannah (2005, 204) offers an explanation for this rather socially unacceptable practice ‘*Whether as a dry powder or as blended into scented oil*, *its purpose must have been to improve the appearance of the skin of the wearer*… *by creating the impression of a sun tanned healthy male without the chore of exercise at the gymnasium*’. Olive oil is a good dispersant (a liquid carrier to prevent agglomeration) for fine iron or lead oxide particles (Arulmozhi and Mythili, 2013); thus Kean *miltos* mixed with olive oil would have spread evenly on the skin to create a good ‘suntan’ effect. In architecture, or for joinery work, *miltos tektoniki* was used, with places of origin, reported, i.e. Egypt and Spain (Dioscorides, V.96).

2 Inscription IG II^2^ 1128 found in the Agora of Athens concerns a decree dated (*c*. 360 BC) issued by Athens to the three city states of Kea (Ioulis, Korisseia and Karthaia (Fig. 1b)) requiring them to export *miltos* in its entirety from their respective mines exclusively to Athens, even bearing the charges for the transport. The tone is severe and there were penalties imposed. This has been considered rather an odd ‘demand’ given that Athens had access to the large iron oxide deposits of Laurion (see Geology section) and therefore the meaning of this inscription has been interpreted in different ways. Lytle (2013) has concluded that Kean *miltos* must have been used in boat maintenance, as an antifouling agent, and to a lesser extent as a decorative paint.

3 Lytle (2013) presents new evidence associated with the use of *miltos* in agriculture. In the late 4th century BC, *miltos* is reported to have been mixed with pitch (*miltopissa*) in an inscription from Eleusis (*IG II*^2^ 463.90) (Lytle 2013, 537). In a 3rd century AD agricultural manual, the author recommends that Lemnian *miltos*, in a paste with water, is smeared around the roots of plants in an attempt ‘*to prevent trees and vines from being harmed by worms or anything else*’ (Lytle, 2013, 538) and in particular fig trees (Lytle 2013, 539); *miltos* is also placed with olive oil lees (*amurgo*) around roots (Lytle 2013, 539). Separate and distinct from root application seems to be the mixing of *miltos* with pitch and applied to tree trunks. This is for protecting trees from ants (Lytle 2013, 540).

4 Theophrastus never suggested that Kean *miltos* was medicinal. The medicinal applications of *miltos* first appear in Dioscorides (*De Materia Medica* V.96) and Pliny (*Natural History* 35.31–5; 33.111–17) who discuss Sinopic and Lemnian *miltos* only. Pliny considers the Lemnian *miltos* far superior. References to Lemnian *miltos* also occur in Nicander (2nd century BC) (*Theriaca* 864–5). Further medicinal applications are included in Aetius Amidenus (first half 6th century AD). ‘*Miltos has properties that are astringent*, *binding and drying accordingly it is mixed in plaster for wounds and used in pills that are dessicative and drying*. *Taken with fried eggs as an enema it checks the bowels*. *Taken as a drink it even drives out worms* (Lytle 2013, 544). The above echoes the description given in Dioscorides (*De Mat Medica* V. 96). Nicander's reference to *Lemnian miltos* and Dioscorides’ *miltos of Lemnos* suggest association with red Lemnian Earth (Photos-Jones et al., 2017). Galen wrote that there were three kinds of Lemnian Earth, only one of which was medicinal. Although all three came from the same ‘pit’, Galen implies that each ‘layer’ of clay within the pit was processed differently resulting in two additional grades, one used as pigment and the other as fulling agent (Caley and Richards, 1956).
